# Pre-operative/Neoadjuvant Therapy and Vascular Debranching Followed by Resection for Locally Advanced Pancreatic Cancer (PREVADER): Clinical Feasibility Trial

**DOI:** 10.3389/fmed.2021.588375

**Published:** 2021-05-24

**Authors:** Ulrich Ronellenfitsch, Christoph W. Michalski, Patrick Michl, Sebastian Krug, Joerg Ukkat, Joerg Kleeff

**Affiliations:** ^1^Department of Visceral, Vascular and Endocrine Surgery, University Hospital Halle (Saale), Martin-Luther-University Halle-Wittenberg, Halle (Saale), Germany; ^2^Department of Internal Medicine I, University Hospital Halle (Saale), Martin-Luther-University Halle-Wittenberg, Halle (Saale), Germany

**Keywords:** pancreatic cancer, preoperative chemotherapy, arterial resection, feasibility trial, multimodal treatment approach

## Abstract

**Introduction:** Pancreatic cancer continues to have a poor outcome. Many patients are diagnosed with advanced disease, and in a considerable proportion, abutment or invasion of visceral arteries is present. Moreover, some patients have anatomical variations or stenosis of major visceral arteries requiring arterial reconstruction upon pancreatic cancer resection to avoid organ ischemia. Simultaneous arterial reconstruction during resection is associated with relevant morbidity and mortality. This trial evaluates the approach of visceral debranching, that is, arterial reconstruction, prior to neoadjuvant chemotherapy and tumor resection in patients with locally advanced, unresectable pancreatic cancer.

**Methods and Analysis:** The trial includes patients with locally advanced, non-metastatic pancreatic cancer with arterial abutment or invasion (deemed primarily unresectable), variations in vascular anatomy, or stenosis of visceral arteries. The participants undergo visceral debranching, followed by current standard neoadjuvant chemotherapy (mFOLFIRINOX, gemcitabine–nab-paclitaxel, or other) and potential subsequent tumor resection. The primary outcome is feasibility, measured as the proportion of patients who start neoadjuvant therapy within 6 weeks of visceral debranching. The trial has an exact single-stage design. The proportion below which the treatment is considered ineffective is set at 0.7 (H0). The proportion above which the treatment warrants further exploration in a phase III trial is set at 0.9 (H1). With a power (1-beta) of 0.8 and a type 1 mistake (alpha) of 0.05, the required sample size is 28 patients. Feasibility of the approach will be assumed if 24 of the enrolled 28 patients proceed to neoadjuvant chemotherapy within 6 weeks from visceral debranching.

**Discussion:** This trial evaluates a new treatment sequence, that is, visceral debranching followed by chemotherapy and resection, for pancreatic cancer with invasion or abutment of visceral arteries. The primary objective of the trial is to evaluate feasibility. Trial results will allow for estimating treatment effects and calculating the sample size of a randomized controlled trial, in which the approach will be tested if the feasibility endpoint is met.

**Clinical Trial Registration:** clinicaltrials.gov, identifier: NCT04136769.

## Introduction

Pancreatic cancer continues to have a poor outcome for patients. One of the reasons is that diagnosis is often established late and already at a locally advanced stage. The only potentially curative treatment for pancreatic cancer is surgical resection (1). A major obstacle to a safe and oncologically successful resection is the abutment or encasement of large visceral arteries, namely, the celiac trunk or the superior mesenteric artery (SMA), by the tumor. Such invasion or abutment is present in up to a third of patients upon diagnosis (2, 3). Moreover, some patients have variations in vascular anatomy with aberrant or aplastic visceral arteries or occlusive disease of the celiac trunk or SMA (4, 5). In such situations, there is exclusive or predominant vascularization of the mesentery or liver *via* collateral vessels, which need to be ligated during tumor resection. Therefore, if complete resection of the tumor is aimed for, arterial reconstruction is required to prevent organ ischemia.

Resection of the tumor, usually carried out as partial pancreatoduodenectomy (Whipple's procedure) or distal or total pancreatectomy, with simultaneous arterial reconstruction is technically possible. In contrast to venous reconstruction, which is necessary in cases of venous tumor invasion, arterial reconstruction bears a considerable perioperative morbidity and mortality, with the latter reaching up to 45% in some series. Moreover, the oncological efficacy of resection with arterial reconstruction is often limited because of microscopically incomplete resection (6–8). In recent years, the concept of neoadjuvant chemotherapy for pancreatic cancer has evolved, thanks to the availability of safe, relatively well-tolerated, and effective combination schemes (FOLFIRINOX, gemcitabine–nab-paclitaxel, and others) (9). For example, in a randomized trial comparing neoadjuvant FOLFIRINOX and gemcitabine–nab-paclitaxel, only nine out of 103 patients did not reach surgery due to the toxicity of the neoadjuvant therapy (10). Yet even after neoadjuvant chemotherapy, visceral artery invasion usually does not resolve completely, thus still requiring arterial reconstruction. In order to avoid the relevant morbidity and mortality associated with simultaneous resection and reconstruction, a split in the therapeutic approach with arterial reconstruction (“visceral debranching”) prior to neoadjuvant chemotherapy and resection seems reasonable.

Here we present the protocol (version 1.2, December 24, 2019) of a clinical trial assessing the feasibility of visceral debranching followed by chemotherapy and resection in patients with locally advanced pancreatic cancer. The protocol has been written and is presented in accordance with the SPIRIT checklist (11).

## Methods and Analysis

### Study Objectives

#### Primary Objective

The primary objective of this trial is to assess the feasibility of visceral debranching prior to neoadjuvant chemotherapy and resection in locally advanced, unresectable pancreatic cancer.

#### Secondary Objectives

The secondary objectives of this trial are as follows:

— to assess the efficacy of visceral debranching prior to neoadjuvant chemotherapy and resection in terms of complete resection of the tumor,
— to assess the safety of visceral debranching prior to neoadjuvant chemotherapy and resection, and
— to evaluate survival in patients undergoing visceral debranching prior to neoadjuvant chemotherapy and resection.

### Endpoints

#### Primary Endpoint

The primary endpoint of the trial is the proportion of patients proceeding to neoadjuvant chemotherapy (at least one dose administered within 6 weeks from the debranching procedure) among all patients undergoing visceral debranching.

#### Secondary Endpoints

The secondary endpoints of the trial are as follows:

— proportion of patients proceeding to attempted tumor resection among all patients undergoing visceral debranching,
— proportion of patients with clear resection margins (R0) upon pancreatic cancer resection following visceral debranching and neoadjuvant chemotherapy among all patients undergoing visceral debranching,
— perioperative in-hospital morbidity associated with the visceral debranching procedure, measured according to the Clavien–Dindo Classification of surgical complications (12),
— toxicity during neoadjuvant chemotherapy, measured according to the Common Terminology Criteria for Adverse Events (CTCAE), version 5.0 (13),
— perioperative in-hospital morbidity and mortality associated with pancreatic cancer resection, measured according to the Clavien–Dindo Classification (12),
— progression-free survival, defined as the time between first diagnosis, which is assumed equivalent to study enrolment, and documented progression according to Response Evaluation Criteria in Solid Tumors criteria, version 1.1 (14),
— recurrence-free survival, defined as the time between resection and the appearance of local recurrence, peritoneal carcinomatosis, or distant metastases. For patients who are not resected, recurrence-free survival will be defined as zero; and
— overall survival, defined as the time between first diagnosis, which is assumed as equivalent to study enrolment, and death, independent of the cause of death.

### Trial Design

The trial is designed as a single-arm multi-center study. It will be conducted at the principal study center [University Hospital Halle, Halle (Saale), Germany] as well as in other study centers with sufficient expertise in pancreatic surgery, vascular surgery, and pancreatic oncology—yet to be defined.

**Figure 1:**
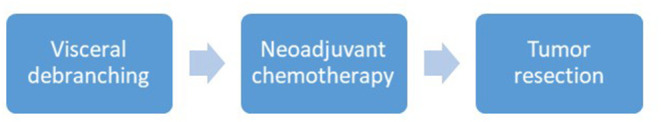
Flow chart of the trial sequence.

The decision for visceral debranching and neoadjuvant chemotherapy is taken, and eligibility for these procedures and subsequent tumor resection is ascertained in a multidisciplinary tumor board. Afterwards, screening of the patient for the remaining inclusion and exclusion criteria takes place. If eligibility for study inclusion is confirmed, the patient is informed about the aims of the study and all study-specific procedures, and asked to provide informed consent (Figure 1).

### Study Population

#### Inclusion Criteria

**Figure 2:**
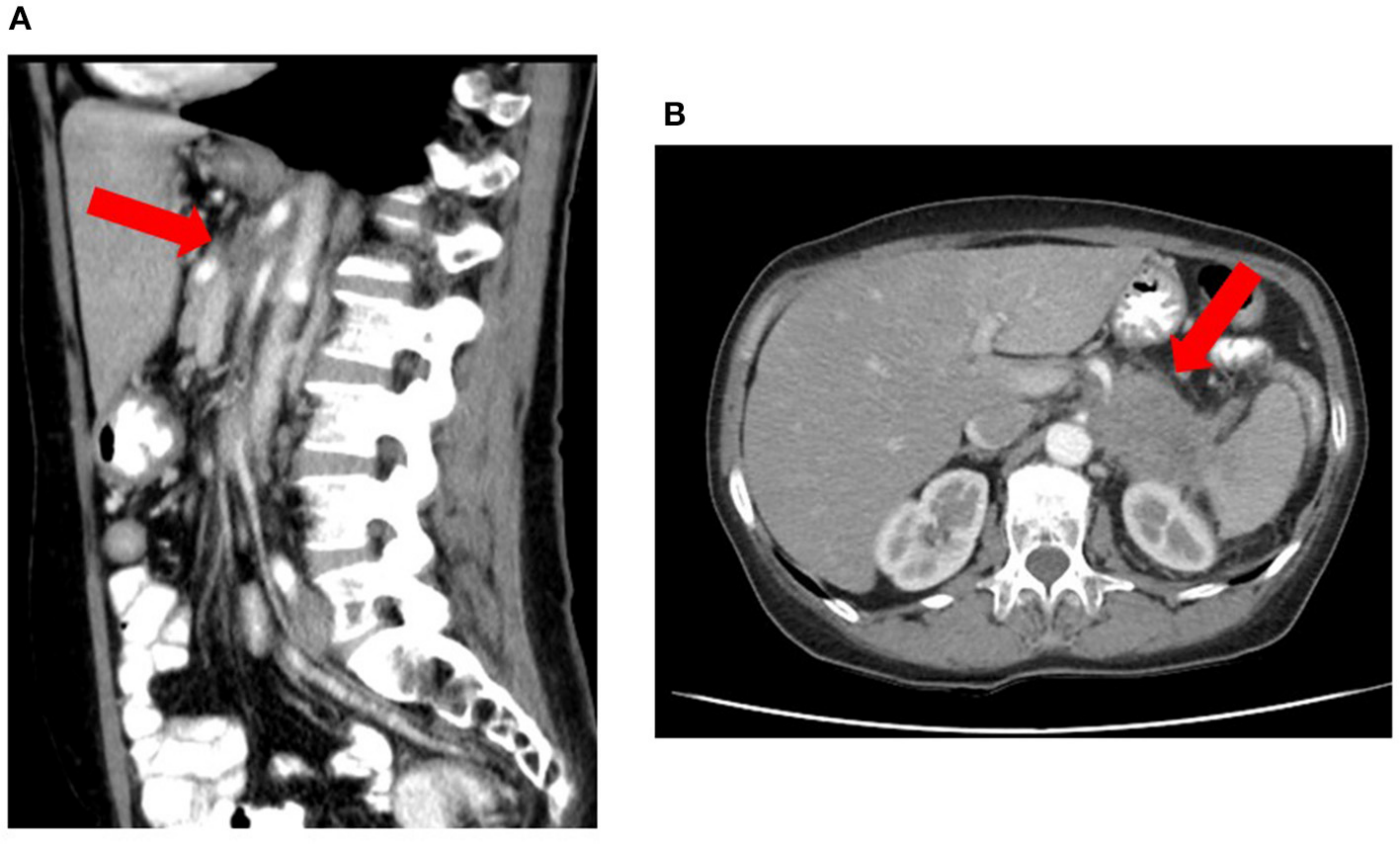
Locally advanced pancreatic corpus adenocarcinoma considered unresectable due to arterial invasion on CT according to National Comprehensive Cancer Network and International Study Group of Pancreatic Surgery criteria. The red arrow delineates the tumor, which shows invasion of the superior mesenteric artery and celiac trunk both on sagittal **(A)** and transversal **(B)** images.

**Figure 3:**
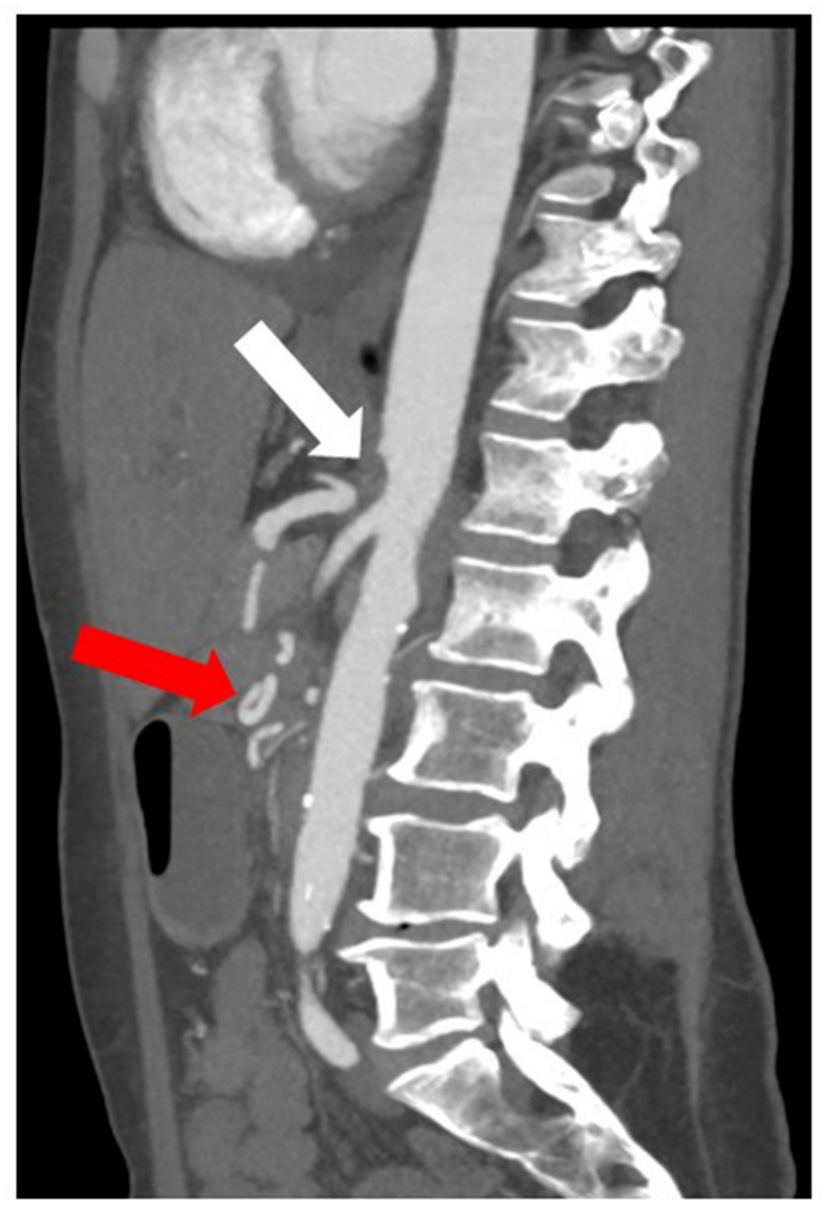
CT image of an anatomic variation of the visceral arteries with aplasia of the origin of the celiac trunk in the aorta (white arrow) and collateralization of the hepatic artery *via* a network of pancreatoduodenal collaterals (red arrow).

— Pancreatic cancer (pancreatic ductal adenocarcinoma, IPMN-derived adenocarcinoma, adenosquamous carcinoma), diagnosed by preoperative biopsy or cytology or intraoperative biopsy during the visceral debranching procedure
— Evidence of locally advanced disease which is considered unresectable due to arterial invasion on CT or MRI (Figure 2) according to National Comprehensive Cancer Network (NCCN) and International Study Group of Pancreatic Surgery (ISGPS) criteria (15, 16):
  - ° Tumor encasement (>180°) of the SMA or celiac trunk
  - ° Tumor encasement (>180°) of a short segment of the hepatic artery
  or
  anatomic variation of the visceral arteries with vascularization of the liver or mesentery *via* collaterals which need to be ligated during tumor resection (e.g., gastroduodenal artery), as shown on CT or MRI (Figure 3).
  or
  high-grade stenosis or occlusion of either the celiac trunk or the SMA with vascularization of the liver or mesentery *via* collaterals which need to be ligated during tumor resection (e.g., gastroduodenal artery), as shown on CT or MRI, which is not amenable to endovascular revascularization
— Invasion of the portal or superior mesenteric vein may be present but must be considered resectable (involvement with distortion or narrowing of the vein or occlusion of the vein with suitable vessel proximal and distal, allowing for safe resection and replacement) according to NCCN and ISGPS criteria (15, 16).
— Provision of written informed consent prior to performance of study-specific procedures or assessments and willingness to comply with treatment and follow-up
— Age ≥18 years.

#### Exclusion Criteria

— Histologically proven peritoneal carcinomatosis (biopsies of macroscopically suspicious findings must be taken at the beginning of the operation and be analyzed immediately by fresh frozen section)
— Histologically proven distant metastatic disease (biopsy of one metastatic site is sufficient)
— Co-morbidities, organ function, or physical status precluding visceral debranching or intensive neoadjuvant combination chemotherapy, as judged by the treating physicians
— Any serious and/or unstable pre-existing medical, psychiatric, or other conditions that could interfere with the patient's safety, provision of informed consent, or compliance with study procedures.

#### Number of Trial Participants

According to the exact single-stage design of the trial, 28 participants will be recruited into the study (see the section on statistics).

#### Recruitment

The participants will be recruited at surgical, gastroenterological, and oncological departments. Dedicated screening for trial participation will be done when the patient is discussed at the multidisciplinary tumor conference.

### Conduct of the Trial

#### Trial Procedures

The temporal sequence of trial procedures is displayed in Table 1. After recruitment into the trial, the patient will proceed to visceral debranching without any further unnecessary delay. Once the patient has sufficiently recovered from the procedure, neoadjuvant chemotherapy should start. Neoadjuvant chemotherapy is not part of the trial protocol but is conducted according to the judgment of the treating oncologist. Surgical re-exploration with the aim of tumor resection should follow approximately 4 to 6 weeks after completion of chemotherapy. Re-staging *via* CT or MRI prior to the planned tumor resection is mandatory.

**Table 1:** Time and events table.

**Required measures**	**Screening/ trial entry**	**Visceral debranching as soon as possible after trial entry**	**During neoadjuvant chemotherapy intended start date 2 to 3 weeks after debranching**	**Tumor resection intended date of operation 4–6 weeks after end of chemotherapy**	**Follow-up 0–36 months after tumor resection**
Verification of inclusion and exclusion criteria	♦				
Informed consent	♦				
CT/MRI	♦			♦ Between end of chemotherapy and resection	♦
Ascertainment of data as detailed in “evaluation and follow-up”	♦	♦	♦	♦	
Ascertainment of primary endpoint			♦ Start of neoadjuvant chemotherapy		
Follow-up exams at the discretion of the treating physician (including Ca 19-9 measurements and cross-sectional imaging as indicated)					♦
Surgery		♦		♦	

*Measures to be performed at the time points marked with ♦*

##### Visceral Debranching

Following laparotomy, the peritoneum and liver will be explored for any findings suspicious of metastasis. Abnormalities must be biopsied and immediately analyzed by fresh frozen sections. If the finding is positive for peritoneal carcinomatosis or distant metastasis, the patient will be excluded from the trial. If no preoperative histological diagnosis of pancreatic cancer has been established, the procedure should continue with a transduodenal or direct biopsy of the pancreatic tumor or with an excisional biopsy of a suspicious peritumoral lymph node. Biopsies must be assessed by fresh frozen sections in order to obtain an intraoperative result. If the diagnosis of pancreatic cancer has been ascertained, the operation continues with visceral debranching.

Visceral debranching, as such, is carried out according to the individual judgment of a board-certified vascular surgeon, who will perform the procedure. The aim of the procedure is to ensure sufficient arterial blood flow to the mesentery and liver after the subsequent procedure, which then comprises resection of visceral arteries. All open vascular procedures can be employed for visceral debranching. Examples are aorto-visceral or iliaco-visceral bypasses using autologous vein or an allogeneic graft or re-insertion of the SMA or celiac trunk into the aorta. Perioperative anticoagulation treatment is also administered according to the individual judgment of the treating vascular surgeon.

In case of cholestasis or gastric outlet obstruction, a deviating procedure (hepaticojejunostomy, gastroenterostomy) can be carried out during the same operation.

In preparation for the ensuing neoadjuvant chemotherapy, a venous port system or alternative vascular access device for the administration of chemotherapy (e.g., Hickman or Broviac catheter) should be implanted during the visceral debranching procedure.

##### Neoadjuvant Chemotherapy

Neoadjuvant chemotherapy is not part of the trial protocol. The specific chemotherapy regimen and its duration are decided individually by the treating physicians, usually in a multidisciplinary tumor board. All efforts must be made that neoadjuvant chemotherapy starts without any undue delay following the debranching procedure. The start date aimed at should be not later than 2 to 3 weeks after the operation.

##### Re-Staging and Tumor Resection

Re-exploration with the aim of tumor resection should be performed 2 to 4 weeks after the completion of chemotherapy. Prior to resection, re-staging and verification of vascular reconstruction patency should be carried out with CT or MRI. In case of newly detected distant metastasis or unequivocal signs of irresectability, the patient should not proceed to surgery, but the continuation of chemotherapy in palliative intent needs to be discussed with the treatment team. In case of newly onset cholestasis or gastric outlet obstruction, palliative surgery (hepaticojejunostomy, gastroenterostomy) can be carried out.

The specific procedure for tumor resection and intestinal tract reconstruction is at the choice of the treating surgeon. It should follow oncological principles and aim at complete removal of the tumor and regional lymph nodes (1). Usually, resection will be done as partial pancreatoduodenectomy with or without distal gastrectomy (Whipple's procedure or pylorus-preserving Whipple's procedure), distal pancreatectomy with splenectomy, or total pancreatectomy with splenectomy.

### Evaluation and Follow-Up

The temporal sequence of evaluation and follow-up is displayed in Table 1.

#### Trial Entry

Upon trial entry, the following information will be assessed:

- Date of birth and sex
- Date of diagnosis
- Tumor type with histology/cytology determination
- UICC and cTNM stage
- ECOG performance status
- ASA status
- CA 19-9 serum level
- Reason for planned visceral debranching [arterial abutment, arterial encasement with length and perimeter (0–360°) of encasement, anatomic variation of the visceral arteries, high-grade stenosis/occlusion of the visceral arteries].

#### Visceral Debranching

At the time of visceral debranching, the following information will be assessed:

- date of debranching procedure
- intraoperative biopsy (yes/no, result)
- type of debranching procedure
- other procedures carried out (port implantation, hepaticojejunostomy, gastroenterostomy)
- duration of surgery
- estimated blood loss
- number of transfused units of packed red blood cells
- intra- and postoperative in-hospital complications according to the Clavien–Dindo Classification (12)
- incidence of postoperative pancreatic fistula according to the ISGPS definition (17)
- incidence of postpancreatectomy hemorrhage according to the ISGPS definition (18)
- incidence of delayed gastric emptying according to the ISGPS definition (19).

#### Neoadjuvant Chemotherapy

The following characteristics of administered neoadjuvant chemotherapy will be assessed:

- start and end date of chemotherapy
- chemotherapeutic agents
- cumulative dose per agent
- number of cycles
- adverse events occurring during and until 30 days after the end of chemotherapy, recorded according to CTCAE, version 5.0 (13)
- CA 19-9 serum level (between the end of chemotherapy and resection).

#### Tumor Resection

At the time of tumor resection, the following information will be assessed:

- date of operation
- type of resection (approach, extent, lymphadenectomy, resection of visceral arteries)
- other procedures carried out (port implantation, hepaticojejunostomy, gastroenterostomy)
- duration of surgery
- estimated blood loss
- number of transfused units of packed red blood cells
- intra- and postoperative in-hospital complications according to the Clavien–Dindo classification
- incidence of postoperative pancreatic fistula according to the ISGPS definition (17)
- incidence of postpancreatectomy hemorrhage according to the ISGPS definition (18)
- incidence of delayed gastric emptying according to the ISGPS definition (19)
- completeness of resection (R0, R1, R2) and smallest distance between resectional margin and vital tumor tissue found in the specimen, together with the localization where it was found
- possible tumor invasion of visceral arteries (abutment, encasement, invasion into vessel wall)
- final histological result (tumor type, ypTNM stage including L, V, and Pn stages, and number of metastatic and totally harvested lymph nodes)
- regression grade of the tumor according to the College of American Pathologists (CAP) and the Evans grading schemes for neoadjuvant chemotherapy without radiation-treated pancreatectomy specimens (20).

#### Follow-Up

Trial participants should be included into regular oncological follow-up with intervals recommended by the treating physicians according to their individual judgment. Adjuvant chemotherapy can be administered according to the individual recommendation by a multidisciplinary tumor board. The trial protocol does not foresee defined intervals for cross-sectional imaging and CA 19-9 serum measurements, which should however be conducted regularly. In case of diagnosis of recurrence, the date of diagnosis will be documented. Histological verification of recurrence should be aimed for, but clinically and biochemically suspected recurrence will also be counted as such. If a trial participant deceases during follow-up, the date of death as well as the cause of death will be ascertained from death certificates or other medical documentation. Follow-up for the purpose of the trial will cover a period of 5 years from the date of first diagnosis, but regular clinical follow-up can continue longer independently from the trial.

For participants dropping out of the trial, all efforts will be made to collect the above-mentioned data if available and applicable.

### Trial Duration

For each participant, the duration of the trial will be a treatment phase from recruitment into the trial until tumor resection. The primary endpoint will be ascertained at the start of neoadjuvant chemotherapy. The maximum duration of follow-up for each participant is 3 years following resection.

The total duration of the trial is expected to be 18 months (recruitment phase) and 54 months (first patient in to last patient out).

If more than four study participants have failed to reach the primary endpoint of proceeding to neoadjuvant chemotherapy (at least one dose administered within 6 weeks from the debranching procedure), the entire trial can be stopped because H0 cannot be rejected anymore given the study design used.

### Statistical Design and Analysis

#### Sample Size

With regard to the primary endpoint, the study uses an exact single stage design with the following underlying assumptions. The proportion of patients proceeding to neoadjuvant chemotherapy after visceral debranching below which the treatment is considered ineffective is set at 0.7 (H0). The proportion of patients proceeding to neoadjuvant chemotherapy after visceral debranching above which the treatment warrants further exploration in a phase III trial is set at 0.9 (H1). With a power (1-beta) of 0.8 and a type 1 mistake (alpha) of 0.05, the required sample size is 28 patients.

#### Analysis of Endpoints

Analysis of the primary endpoint takes place as soon as all patients have undergone visceral debranching and completed neoadjuvant chemotherapy and surgery with the intent to resect the tumor or were unable to proceed to these treatments after visceral debranching. The primary endpoint will be presented as a proportion with 95% confidence interval. H0 will be rejected and feasibility of the approach under study will be assumed if 24 of the enrolled 28 patients proceed to neoadjuvant therapy within 6 weeks from the debranching procedure.

All secondary endpoints will be analyzed descriptively. The secondary endpoint proportion of patients with clear resection margins (R0) upon pancreatic cancer resection following visceral debranching and neoadjuvant chemotherapy among all patients undergoing visceral debranching will be analyzed at the same time as the primary endpoint. Perioperative in-hospital morbidity and mortality associated with the visceral debranching procedure and pancreatic cancer resection will be analyzed once the patient is discharged from the hospital after the respective procedure. The incidence of complications will be presented as proportion with 95% confidence interval, stratified by the highest Clavien–Dindo grade of all complications that occurred in a given patient. Toxicity of neoadjuvant chemotherapy will be analyzed 30 days after completion of chemotherapy. The incidence of adverse events will be presented for the safety population, defined as patients who received at least one dose of chemotherapy. For each type of adverse event, the worst grade observed across the whole therapy will be tabulated, and the percentages of grade 2+ and grade 3+ cases will be provided.

The secondary endpoints progression-free, recurrence-free, and overall survival will be analyzed when data are mature, that is, when the respective median survival has been reached. Survival curves will be estimated with the Kaplan–Meier method and displayed graphically.

### Data Management and Data Protection

The trial will be conducted in full accordance with medical confidentiality and the provisions of the German Federal Data Protection Act as well as the European Union's General Data Protection Regulation.

On giving their consent to participate in the trial, the patients agree that their study-related data are recorded in pseudonymous form. The pseudonymization key (patient identification list) will be generated so that no conclusion on the identity of the specific individual can be drawn. It will be kept strictly separate from all data. All data and the pseudonymization key will be stored in a secured manner. Paper-based data will be kept in a locked container. Electronic data will be stored in password-secured files on secure servers of the study center. Keys and passwords will be made available exclusively to personnel directly involved with the conduct and analysis of the study. The data and the pseudonymization key will be stored for the duration of the trial and 10 years thereafter, and these will subsequently be deleted.

The originals of all central study documents are to be archived for at least 10 years after the end of the trial. The principal investigator retains the generated administrative documents (correspondence with the ethical committee, etc.), patient identification list, signed informed consent forms, and copies of the general study documentation (protocol, amendments) for the period stated above. The trial participants have the right to request deletion of their stored individual data any time throughout the trial unless there is a legal requirement to retain the data.

The participants' informed consent includes that data may be forwarded to other researchers for secondary analysis only in anonymized form upon request for specific secondary analyses.

After due consideration, all participating investigators are convinced that the trial has a favorable risk–benefit ratio with regard to data management and data protection. The benefits of the accrual and analysis of patient data, which will be pseudonymized and stored in a defined way respecting established data protection standards, outweigh the associated potential risks for the trial participants.

## Ethics and Dissemination

### Patient and Public Involvement

The patient organization Arbeitskreis der Pankreatektomierten e. V. (www.bauchspeicheldruese-pankreas-selbsthilfe.de; German patient support group for patients with diseases of the pancreas) was involved and provided valuable advice during the planning of the trial and the writing of the trial protocol. Upon trial completion and availability of results, patient involvement will be sought to disseminate the results within the patient community and the public.

### Ethical Considerations and Regulatory Issues

All participating investigators are convinced that the trial has a favorable risk–benefit ratio. Tumor resection is the only potentially curative treatment in pancreatic cancer and offers a relevant survival benefit (21). In tumors invading major visceral arteries, resection can only be performed if the invaded arteries are resected and reconstructed. Neoadjuvant chemotherapy is commonly used in locally advanced tumors, with the aim of downsizing the tumor and facilitating complete resection (21). Both neoadjuvant chemotherapy and arterial reconstruction during resection are considered established treatments for locally advanced pancreatic cancer in selected patients. The novel approach of this trial consists of performing arterial reconstruction separately from tumor resection. The expected benefit is lower perioperative morbidity and mortality. Performing arterial reconstruction and tumor resection in a one-stage approach is associated with prohibitive perioperative morbidity and mortality, with the latter exceeding 10% in several series (8). The general risks associated with an additional operation, such as anesthesia-associated risks, bleeding, wound infections, or subsequent ventral hernia, are expected to be substantially lower than the expected benefit in terms of morbidity and mortality reduction. There is the theoretical risk of releasing tumor cells upon manipulating the tumor, for example, for biopsy, during the first operation. However, if one assumes that tumor manipulation releases tumor cells, this will inevitably happen in an identical manner during a one-stage procedure comprising both arterial reconstruction and tumor resection or during biopsy preceding neoadjuvant chemotherapy. Therefore, no incremental risk of tumor cell dissemination is assumed from trial participation.

Separating vascular debranching from resection inevitably leads to a delay in the initiation of neoadjuvant chemotherapy compared to patients in whom chemotherapy is the first step of treatment. The study protocol foresees chemotherapy to begin as soon as possible after debranching. The anticipated time interval is 2 to 3 weeks, but conditions such as perioperative complications could preclude the initiation of chemotherapy and prolong this interval. During this time, tumor progression or metastasis is conceivable. However, although an individual prediction of the velocity of tumor progression is not reliably possible, the interval is deemed sufficiently short not to be associated with a relevant risk of tumor progression. An undue delay of chemotherapy initiation would be detected through the study design, in which the safety outcome failure to start chemotherapy timely enough after diagnosis is the primary endpoint. The chosen primary endpoint mirrors the feasibility of the novel treatment approach or, in other words, the ability to apply vascular debranching as a novel treatment without putting the patient at risk by delaying the current standard treatment. It is thus appropriate to answer the underlying research question.

All complications, both intra- and postoperative, are documented and reported to the principal investigator. He can interrupt the trial at any point, or, in accordance with the ethical committee, implement changes to the study protocol.

The study is conducted in accordance with the applicable version of the declaration of Helsinki. Prior to study initiation, approval from the principal study center's ethical committee has been sought [Ethics Committee of the Medical Faculty of the Martin-Luther-University Halle-Wittenberg, Halle (Saale), Germany, 2019-152]. Approval from competent ethical committees of other participating centers will be sought prior to their initiation. Before enrolment into the trial, all patients are informed in writing (online supplementary material) and verbally, by one of the investigators, about the nature and implications of the trial and especially about the possible benefits and risks for their health. The patients document their consent by signing the informed consent form. The patients can leave the trial at any point without providing a reason for doing so. In this case, treatment of the patient will continue according to the individual judgment of the treating physicians.

Given that visceral artery revascularization and pancreatic resection are considered routine surgical treatments and given that the trial evaluates the novel therapeutic sequence rather than a novel procedure, there is no requirement for a trial-specific patient insurance. The trial participants are insured by the respective hospital's insurance covering inpatient treatments.

The trial has been registered in a publicly available repository for clinical trials prior to initiation comprising all items of the World Health Organization Trial Registration Data Set (clinicaltrials.gov, NCT04136769).

All planned substantial changes will be submitted for approval to the competent ethical committees as protocol amendments and communicated to the publicly available repository as well as all investigators.

## Dissemination Strategy

It is aimed to publish the trial results in the form of one or several manuscripts in peer-reviewed international scientific journals. The principal investigator will review all manuscripts to prevent forfeiture of patent rights to data not in the public domain. The authorship list will be agreed on by the principal investigator prior to publication. Investigators from all study sites will be offered authorship on manuscripts according to the number of patients included in the study. It is not planned to use a professional writer. Publication of the first manuscript reporting the study results is planned to take place as soon as possible after analysis of the primary endpoint. Efforts are made that the pertinent manuscript is not submitted later than 6 months after the results are available.

## Discussion

The study has been purposely designed as a feasibility trial. This study design comes along with a number of limitations. The sample size of the study has been calculated based on its primary endpoint, the timely initiation of neoadjuvant chemotherapy, which is a direct indicator of feasibility. It is inevitable that this sample size does not yield sufficient statistical power for the analysis of secondary endpoints such as efficacy endpoints. The analyses of these endpoints are merely exploratory. If the primary endpoint is met and feasibility is shown, the current secondary endpoints will be formally assessed in an ensuing confirmatory trial, in which overall survival as the most meaningful oncological endpoint would be chosen as the primary outcome.

In designing the trial, it was decided not to consider neoadjuvant chemotherapy an actual study treatment and not to stipulate its details in the protocol. The aim was to grant therapeutic freedom to treating physicians in their choice of the specific chemotherapy scheme. Furthermore, it would have been unusual to consider a treatment which takes place after assessment of the primary endpoint a defined study treatment.

Rather than using strict inclusion and exclusion criteria for age, results from diagnostic and laboratory exams, and risk scales, for this feasibility trial, we rely on the judgment of the treating physicians, that is, surgeons and gastrointestinal oncologists. They are to assess patients regarding co-morbidities, organ function, or physical status precluding vascular debranching or intensive neoadjuvant combination chemotherapy and exclude those not deemed eligible for the procedures. This approach enhances the external validity of the study.

In conclusion, this trial is designed to evaluate the feasibility of a novel treatment sequence, that is, visceral debranching followed by chemotherapy and then resection, for locally advanced pancreatic cancer with invasion of visceral arteries. The trial design has been tailored for this purpose. If the primary endpoint feasibility is met, a subsequent confirmatory randomized controlled trial, in which the efficacy of the approach is tested, will be carried out.

## Ethics Statement

The studies involving human participants were reviewed and approved by Ethics Committee of the Medical Faculty of the Martin-Luther-University Halle-Wittenberg, Halle (Saale), Germany. The patients/participants provided their written informed consent to participate in this study.

## Author Contributions

JK and UR conceived of the trial idea and overall trial design. UR drafted the first version of the trial protocol, edited its final version, planned the trial statistics, and wrote the corresponding sections of the protocol. JK, UR, and CM planned and designed the general and pancreatic surgery-related aspects of the trial and wrote the corresponding sections of the protocol. PM and SK planned and designed the pancreatic oncology-related aspects of the trial and wrote the corresponding sections of the protocol. JU planned and designed the vascular surgery-related aspects of the trial and wrote the corresponding sections of the protocol. All the authors critically reviewed and amended the entire trial protocol and approved of its final version and agree to be accountable for all aspects of the work in ensuring that questions related to the accuracy or integrity of any part of the work are appropriately investigated and resolved.

## Conflict of Interest

The authors declare that the research was conducted in the absence of any commercial or financial relationships that could be construed as a potential conflict of interest.

## Abbreviations

CAP: College of American Pathologists
CTCAE: common terminology criteria for adverse events
ISGPS: International Study Group of Pancreatic Surgery
NCCN: National Comprehensive Cancer Network
RECIST: response evaluation criteria in solid tumors
SMA: superior mesenteric artery
SPIRIT: standard protocol items: recommendations for interventional trials.
